# Enhancement of Collagen-I Levels in Human Gingival Fibroblasts by Small Molecule Activation of HIF-1α

**DOI:** 10.1021/acs.jafc.2c09059

**Published:** 2023-05-03

**Authors:** Lucia Adriana Lifshits, Miryam Rabin, Ran Tohar, Francesca Netti, Matan Gabay, Marina Sova, Daniel Z. Bar, Evgeny Weinberg, Lihi Adler-Abramovich, Maayan Gal

**Affiliations:** Department of Oral Biology, The Goldschleger School of Dental Medicine, Faculty of Medicine, Tel Aviv University, Tel Aviv 6997801, Israel

**Keywords:** collagen, hypoxia, extracellular matrix, hypoxia-inducible factor (HIF), fibroblasts

## Abstract

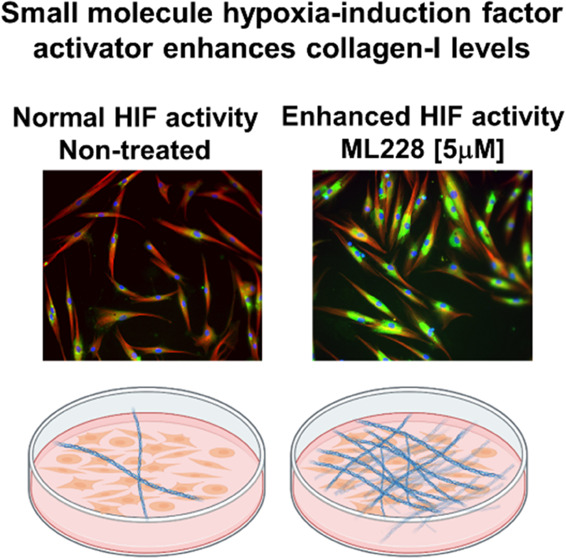

Collagen is the most abundant protein in various mammalian tissues and has an essential role in various cellular processes. Collagen is necessary for food-related biotechnological applications such as cultivated meat, medical engineering, and cosmetics. High-yield expression of natural collagen from mammalian cells is challenging and not cost-effective. Thus, external collagen is obtained primarily from animal tissues. Under cellular hypoxia, overactivation of the transcription factor hypoxia-inducible factor (HIF) was shown to correlate with enhanced accumulation of collagen. Herein, we showed that the small molecule ML228, a known molecular activator of HIF, enhances the accumulation of collagen type-I in human fibroblast cells. We report an increase in collagen levels by 2.33 ± 0.33 when fibroblasts were incubated with 5 μM of ML228. Our experimental results demonstrated, for the first time, that external modulation of the hypoxia biological pathway can boost collagen levels in mammalian cells. Our findings pave the way for enhancing natural collagen production in mammals by altering cellular signaling pathways.

## Introduction

The triple helix collagen is the most abundant protein in animals. It is found primarily in connective tissues, such as skin, tendons, and bones, and is a major protein within the extracellular matrix (ECM).^1,2^ The majority of collagen is transcribed and secreted from fibroblast cells that support various organs. Its fibrous nature is key to maintaining the mechanical properties of the ECM, enabling its physiological functions.^3^ Thus, collagen is considered an essential protein in health and disease, a therapeutic protein in medicine,^4,5^ and a central skeletal protein for multiple biological applications in the pharmaceuticals, cosmetics, and food industries.^6,7^ The primary source of collagen for biomedical applications is bovine collagen type-I. However, there is a growing demand for collagen from non-animal sources, especially for producing cultured meat.^8^ In the latter, collagen and gelatin are used as scaffold materials that support tissue engineering of the cells. Animal-derived polymers like collagen are often regarded as the gold standard for producing scaffolds with ECM-like properties. However, the use of animal ingredients for cultured meat production contradicts the purpose of non-animal meat. Therefore, animal-free scaffolds are being investigated as potential sources of stable, chemically defined, low-cost materials for cultured meat production.

The need for alternative collagen sources has yielded intensive efforts to recombinantly express proteins in plants and microorganisms.^9−15^ The expression of recombinant collagen has several advantages, such as the ability to highly purify the resulting protein and genetically manipulate the amino acid sequence of the expressed collagen. However, various challenges related to collagen’s solubility, immunogenicity, and functionality have hampered the large-scale production and application of collagen from such sources.^16^ Among these, specific post-translational modifications such as the hydroxylation of prolines along the primary sequence of collagen are crucial for obtaining functional collagen. Indeed, hydroxyprolines constitute more than 10% of the amino acid content of the collagen molecule and contribute to the proper folding, packing, and stability of collagen,^16^ and the regulation of interactions with other ECM proteins.^17^ An additional approach is mimicking collagen fibers using a minimalistic peptide sequence of the collagen repeating unit Gly–Pro–Hyp. This was shown to display left-handed polyproline II super-helical packing as in the native single-strand collagen, and the coassembly of such peptides with low molecular weight hydrogelator leads to the formation of stable scaffolds.^18^ The biosynthesis of natural collagen from mammalian cells that contain all of the required elements for proper post-translational modifications has also been explored.^19−21^ However, these efforts resulted in a relatively low yield and high-cost production.

ECM composition, including collagen level, is affected by various cellular conditions. Among these, oxygen level is vital to determining the functionality and characteristics of the ECM.^22−24^ Low cellular oxygen levels (i.e., hypoxia) can arise from external factors such as high altitude or from biological conditions such as wounds, inflammation, and tumors.^25,26^ The cellular response to hypoxia involves activating a large number of genes. Among these, the hypoxia-inducible factor (HIF) transcriptional regulator family is a critical component in response to hypoxia.^27,28^ Under normal oxygen levels (i.e., normoxia), HIF activity is heavily regulated by HIF-prolyl hydroxylases (PHDs). These enzymes hydroxylate specific prolines along the primary sequence of HIF.^29^ This essential interaction tags HIF as a substrate for the von Hippel–Lindau (VHL) E3-ligase complex, which ubiquitinates HIF and targets it for degradation in the proteasome.^30−32^ PHD-mediated hydroxylation is oxygen-dependent. Thus, hypoxia hampers the hydroxylation of HIF by PHD, promoting rapid accumulation of HIF and accelerated transcription of target genes under the control of hypoxia-responsive element (HRE) promoters. HIF activity affects various cellular functions and properties, including ECM composition, and is directly related to collagen levels.^33−35^ Among the diverse tissues in which HIF plays an imperative regulatory role are the gingiva and the periodontal ligament. Hypoxia was shown to significantly upregulate the accumulation of collagen type-I in such tissues.^36^

As HIF overactivity enhances collagen levels, targeted activation of HIF by external modulators under normoxia conditions could lead to overaccumulation of collagen. A number of small molecules and peptides that enhance HIF activity have been identified as inhibitors of HIF hydroxylation.^37−41^ One example, ML228, was identified as an activator of the HIF pathway by a pioneered screening of small molecules that are part of the molecular libraries repository of the National Institute of Health.^42^ The chemical structure of ML228 is based on a triazine molecular scaffold that yields an EC50 of about 0.5 μM in U20 cells. Herein, we explored the ability of ML228 to enhance the accumulation of collagen type-I in fibroblast cells based on the activation of the HIF pathway.

## Materials and Methods

### Reagents

ML228 with purity >98% was purchased as a powder (Tocris Bioscience) and dissolved in dimethyl sulfoxide (DMSO) to a stock solution of 50 mM. The final DMSO concentration in the cells was 0.04% for an ML228 concentration of 20 μM. The following antibodies were used: collagen-I (ab138492, Abcam), HIF-1α (ab179483, Abcam), β-tubulin (05-661, AP124P Merck), HRP-conjugated secondary antibody goat anti-rabbit/mouse IgG (12–348 Merck), Goat Anti-Rabbit IgG H&L (Alexa Fluor 488 (ab150077, Abcam)), Goat Anti-Mouse IgG H&L (Alexa Fluor 647) (ab150115, Abcam), and DAPI Staining Solution (ab228549, Abcam). Cellular viability was assessed using a live/dead viability assay (04511, Merck) and CellTiter (G7570, Promega).

### Institutional Review Board Statement

The experiments involving primary human cells were approved by the Tel Aviv University Institutional Review Board (IEC No. 0001006-1).

### Cell Culture

Human fibroblasts derived from masticatory mucosa were isolated and cultured as previously described.^43^ Briefly, fragments of masticatory mucosa (from the posterior hard palate) were harvested from three female Caucasian donors, aged 20–30 years, during periodontal procedures. Shallow tissue samples (without submucosa) with a diameter of ∼2 mm were excised using a #15C scalpel blade. Exclusion criteria for potential tissue donors were smoking, any systemic disease, pregnancy or lactation, or a history of periodontal disease. Tissue fragments were separated into connective tissue and epithelium by a 2-h incubation with dispase II (2 mg/mL, Sigma-Aldrich, Rehovot, Israel) at 37 °C. The connective tissue of all three samples was cut into smaller pieces and incubated in a standard culture medium (Dulbecco’s modified Eagle’s medium (DMEM)) with 10% fetal calf serum, 2 mM glutamine, 100 U/mL penicillin, and 100 mg/mL streptomycin. Cell viability was assessed manually by trypan blue dye exclusion (Biological Industries). First to third passage cells were used only if they had a typical fibroblastic morphology and a cell viability level >95%.

### Cell Staining and Immunofluorescence

For microscopy images, cells were cultured on round-glass coverslips in a 24-well plate or in a 96-well glass bottom plate (P96-1.5H-N, Cellvis) and incubated with medium supplemented with the indicated concentrations of ML228 at 37 °C under 5% CO_2_. Cells were then washed additional three times with cold phosphate-buffered saline (PBS), following a 20 min fixation in a 4% paraformaldehyde (PFA) in PBS and briefly washed three times with PBS to remove the PFA, followed by a 20 min wash with blocking and penetration solution (PBS with 0.1% Triton X-100 and 2% BSA). Fixed cells were incubated with antibodies for 1 h at 25 °C, washed, and developed together with fluorescence-tagged secondary antibodies (Alexa 488 and 647, Abcam). Cell nuclei were stained with 1 mg/mL 4′,6-diamidino-2-phenylindole (DAPI Staining Solution ab228549, Abcam). Images were taken using a ZEISS LSM 900 in confocal mode, using Zen 3.1. Fluorescence readouts were obtained at ex/em of 358/461, 495/519 nm, and 652/668 nm for blue, green, and red, respectively.

### Cells Viability and Live/Dead Analysis

Cells were incubated in a medium supplemented with the indicated concentrations of ML228 for 48 h. Fluorescein diacetate 6.6 μg/mL and propidium iodide 5 μg/mL (Merck, NJ) were prepared in DMEM and added to the cells. Confocal images were captured using ZEISS LSM 900. Fluorescence readouts were obtained at ex/em of 490/515 and 535/617 nm for green and red, respectively. Cellular ATP levels were quantified using the CellTiter assay (Promega).

### Image Analysis

All of the images were analyzed using Fiji 2 software (National Institute of Health). Equal field areas were selected for analysis. Quantification of fluorescence signal intensity was performed by color threshold and normalized against β-tubulin intensity. Total collagen was evaluated by total fluorescence area intensity (FLI).

### Western Blot Analysis

Cells at a density of 50 000 cells/well were cultured in a 24-well plate for 2 days, with variable ML228 concentrations. Cells were sonicated and lysed in a buffer containing 20 mM Tris-HCL, pH = 7.5, with protease inhibitor (539134, Merck). Equal amounts of total protein were loaded on a 6% SDS gel and transferred to nitrocellulose membranes that were further blocked with 5% skim milk and incubated overnight with primary antibodies toward HIF-1a and β-tubulin. Detection of collagen-I was done in 6% native gel, enabling observation of nondenatured structural collagen and preventing thermal degradation of the heat-sensitive collagen. After extensive washing with tris-buffered saline supplemented with 0.1% Tween-20 (TBST), the membrane was further incubated with an HRP-conjugated secondary antibody for 1 h. Chemiluminescence values were read following addition of ECL substrate in a ChemiDoc imager (Bio-Rad, CA).

### Statistical Analysis

Statistical comparisons were performed by an ordinary one-way ANOVA as implemented in GraphPad Prism with Dunnett’s post-hoc statistical hypothesis. Each measurement was analyzed against the nontreated sample. For statistical analysis, significance was set as *0.01 ≤ *p* < 0.05; **p < 0.01; ***p < 0.001; and ****p < 0.0001.

## Results

**Figure 1:**
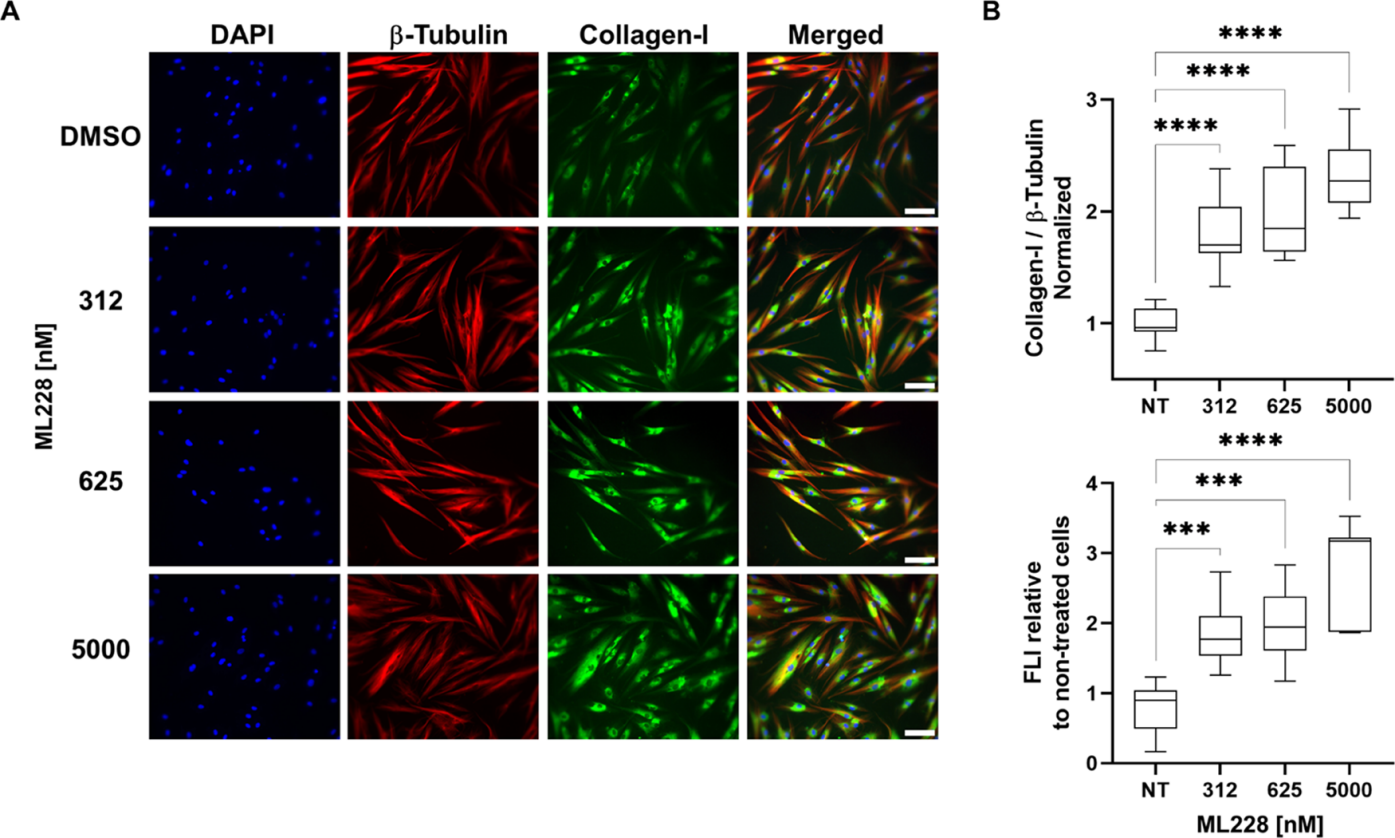
Effect of ML228 on collagen-I levels in HGF cells. Cells were cultured for 48 h with variable concentrations of ML228 and then stained for collagen-I, β-tubulin, and DNA (green, red, and blue, respectively). (A) Representative images of HGF cells. Scale bar: 100 μm. (B) Mean fluorescence intensity relative to nontreated (NT) cells indicating total collagen-I (bottom) and normalized against β-tubulin (top).

Given the correlation between hypoxia and collagen levels detected in fibroblast cells,^36^ we added ML228 to explore the possibility of enhancing collagen-I levels in these cells. In the first step, we incubated the fibroblast cells with varying concentrations of ML228 for 48 h and evaluated collagen-I levels by immunofluorescence. Figure 1A shows representative confocal images that visualize DNA, β-tubulin, and collagen-I in fibroblast cells. Figure 1B shows the corresponding box plot of the mean fluorescence intensity (FLI) of total collagen-I (bottom) and relative to β-tubulin (top) as a function of ML228 concentration. After treatment with ML228, already at a concentration above 312 nM, collagen levels were more than 2-fold higher compared to the baseline of nontreated cells, with maximum collagen levels observed at a concentration of 5000 nM. However, at a higher concentration, collagen levels gradually decreased (see Figure S1A). The effect of ML228 was also tested on an additional cell line, MG-63, an osteoblasts-like cell. Figure S2A shows representative confocal images of the MG-63 cells following incubation with ML228. Figure S2B shows relative collagen type-I levels in nontreated cells and in cells treated with variable ML228 concentrations.

**Figure 2:**
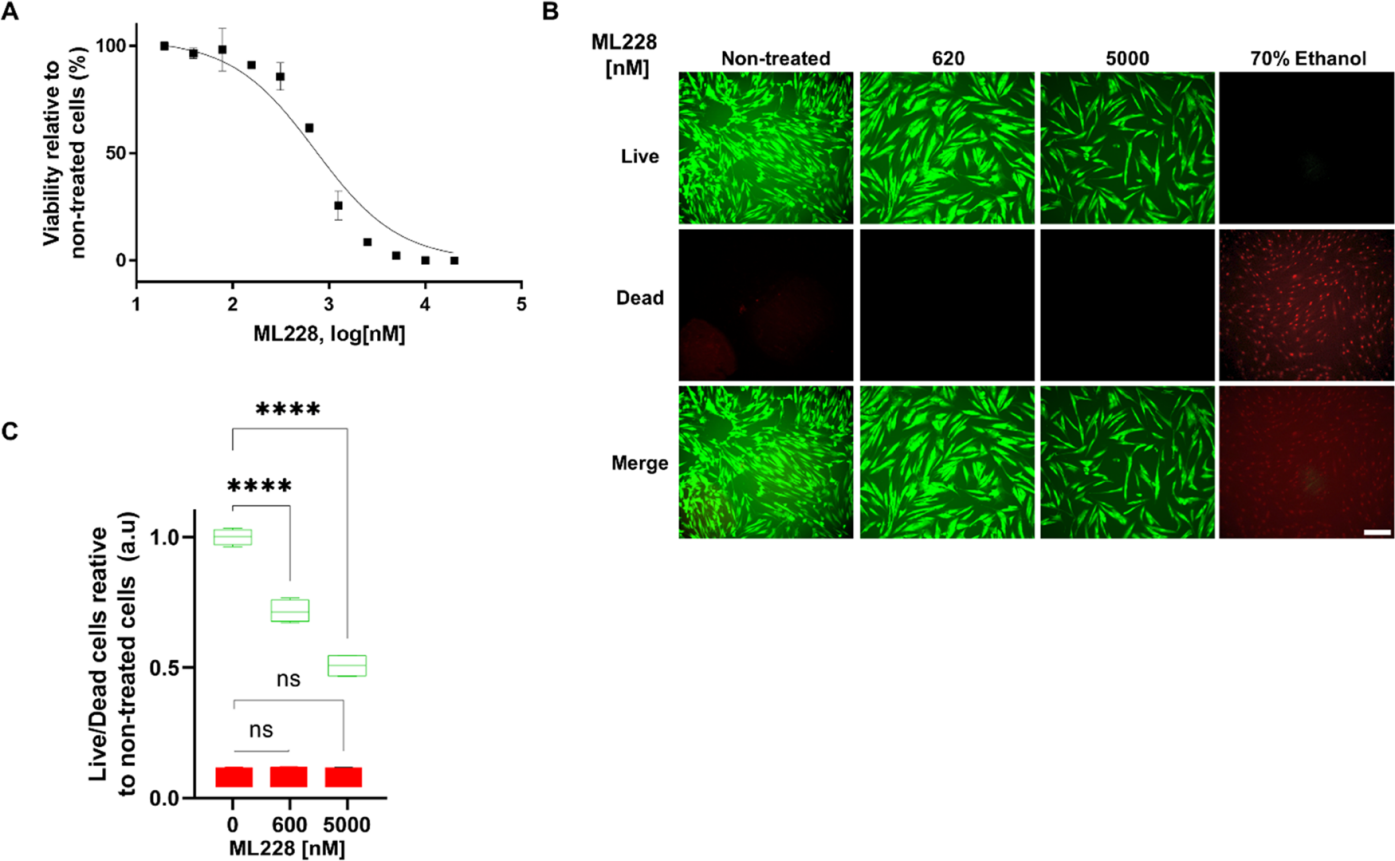
Cellular viability and toxicity. (A) Fibroblasts were incubated with variable concentrations of ML228 for 48 h, and cellular viability was evaluated using the CellTiter assay that measured cellular ATP levels. The curve shows the percentage of live cells relative to nontreated cells, yielding an IC_50_ of 679 nM. (B) Representative confocal images of cells treated with variable concentrations of ML228. Live cells (top row—green), dead cells (middle row—red), and merged (bottom row). Cells treated with 70% ethanol (right column) as a reference for dead cells (right panel). Scale bar: 200 μm. (C) Fluorescence intensity of green and red color is shown relative to nontreated cells.

To gain a better understanding of the pattern of collagen accumulation following treatment with ML228, we studied the latter effect on cellular viability and proliferation. We examined fibroblast viability by evaluating ATP levels following treatment with variable concentrations of ML228 for 48 h. Figure 2A shows a luminescence-based readout measuring the amount of cellular ATP relative to nontreated cells. The calculated IC_50_ of 679 nM is in congruence with the observed levels of collagen-I, suggesting that either molecular toxicity or the inability of the cells to proliferate above a specific threshold concentration hampers additional collagen accumulation. To differentiate between these possibilities, we examined the mechanism by which ML228 affects fibroblast viability. Accordingly, we applied the live/dead assay, which yields distinct green and red colors for live and dead cells, respectively. Figure 2B shows representative images of fibroblast cells treated with ML228 at concentrations of 0, 620, and 5000 nM. Almost no dead cells were observed, indicating that ML228 negatively regulates cell growth without imparting cellular death. Figure 2C shows the relative viability of the cells compared to nontreated cells. Figure S2C shows confocal images of MG-63 cells treated with representative concentrations of ML228 and analyzed with the live/dead assay (Figure S2D).

**Figure 3:**
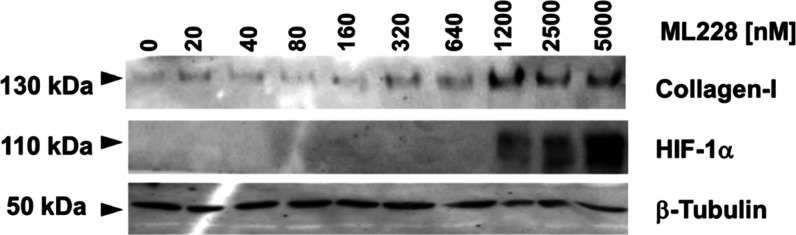
Western blot analysis showing HIF-1α, collagen-I, and β-tubulin protein levels as a function of variable ML228 concentrations. Fibroblast cells were incubated with variable concentrations of ML228 for 48 h, and the cellular lysate was analyzed.

Having established that ML228 induces overaccumulation of collagen-I and does not impart cellular toxicity, we sought to validate that ML228 is indeed correlated with higher levels of HIF-1α. To this end, we performed a Western blot (WB) analysis of collagen-I and HIF-1α levels following the treatment of fibroblast cells with variable concentrations of ML228 (Figure 3). Of note, a major difference between the WB analysis and that performed in Figure 1 is that WB was evaluated on total cellular lysate, including intracellular content. In nontreated cells and in cells treated with low doses of ML228, no HIF-1α protein was observed, indicating that HIF-1α is in its basal levels. However, at higher concentrations of ML228, enhanced levels of HIF-1α were detected, reaching a maximum at a concentration of 5000 nM. A similar pattern was observed for collagen-I, with maximal levels obtained above 1200 nM. This behavior is correlated with the highest levels of collagen-I observed in the confocal images (Figure 1).

## Discussion

**Figure 4:**
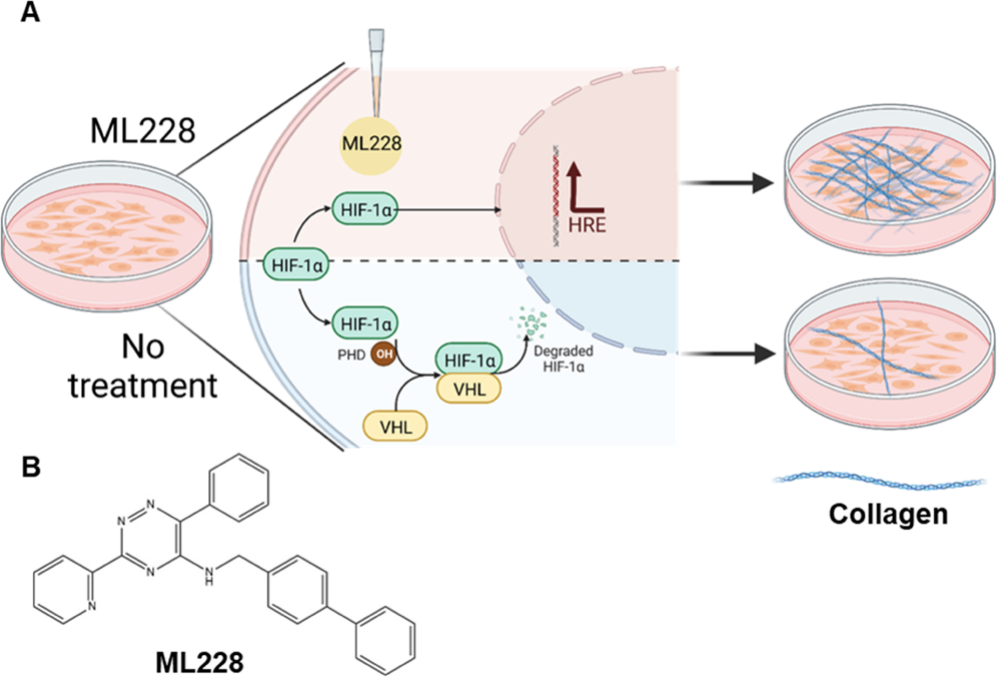
Schematic illustration of Ml228 mechanism of action. (A) Illustration of collagen accumulation via the application of ML228. Under normoxia and without treatment of ML228, the activity of hydroxylated HIF is regulated via its degradation. The application of ML228 leads to higher HIF activity and accumulation of collagen. (B) Chemical structure of ML228.

The evidence that hypoxia strongly affects the ECM in various health and disease conditions,^44−46^ and the evidence that reduced oxygen levels are correlated with increased collagen synthesis and accumulation,^36,47^ can promote improved collagen production. Indeed, such evidence concurs with the importance of high levels of collagen in wounds and inflammation and with the healing processes of these pathologies. Such processes induce a hypoxia-like environment that impairs the natural balance in the synthesis of collagen. Here, we harnessed the hypoxia pathway and showed that modulation of HIF-1α activity by the small molecule ML228 leads to a substantial increase in collagen-I levels in fibroblast cells. We showed that imparting an artificial hypoxia-like state, which enhanced HIF-1α activity, increased collagen accumulation relative to untreated cells. Figure 4 illustrates the experimental scheme and mechanism by which the application of ML228 activates the hypoxia pathway, leading to HIF nuclear translocation and resulting in enhanced collagen accumulation.

Notably, the ML228 mechanism is not clearly defined, and the PHD enzyme may not be the direct target of the molecule. Thus, pinpointing the exact mechanism that leads to HIF activation and collagen overaccumulation is challenging. In addition, the cellular viability of ML228-treated cells showed an IC50 of ∼600 nM. This relatively high value raises the possibility that collagen accumulation may be limited by off-site effects that prevent cellular proliferation. This suggests that the maximal collagen level reported here may not be an inherent characteristic of the hypoxia cellular pathway. Accordingly, the design of more specific and potent molecules, and the exploration of additional inhibitors of proteins that regulate HIF, may yield even higher collagen-I levels.^48,49^ Additional issues to be considered are the effect of activating the hypoxia pathway and the application of small molecule HIF activators on various ECM components, including other collagens. Although type-I is the major collagen type used in food biotechnology applications, understanding the effect of activating the hypoxia pathway on additional collagen types (e.g., type II, III, etc.) is important. Moreover, the effect of ML228 and of other types of HIF activators should be explored in additional cell types. As shown in Figure S2, collagen levels increased also in osteoblast MG-63 cells that were treated with ML228. As the HIF pathway is highly conserved, similar patterns may be expected in other cellular systems. Such exploration could lead to a more potent cellular system for producing mammalian collagen and other ECM factors that are required for food and agricultural applications. In addition to interference with the hypoxia pathway, synergy with other approaches should be explored. An example of this approach is the addition of ascorbic acid (i.e., vitamin C), which is known to stimulate the biosynthesis of procollagen or glycolic acid. Such mixtures could be optimized to further boost collagen levels. An alternative solution, aimed to increase the activity of HIF-1α by interfering with its interaction with PHD, is to harness proteolysis targeting chimeric (PROTAC) modulators, which impart fast degradation of a target protein.^50^ Such a possibility could facilitate PHD degradation and thus maintain high levels of HIF. Finally, several approaches can be synergistically integrated to further increase collagen levels in mammalian cells. Our study demonstrated that modulation of a specific cellular pathway can enhance collagen production in cells. Indeed, the ML228 molecule is designed as an HIF-1α activator that boosts collagen accumulation in fibroblast cells. Due to the essential role of collagen in various health and disease conditions, enhancing its accumulation has immense implications, including in a broad array of biotechnological applications and in the production of animal-free collagen for cultured meat.

## Data Availability

All of the data generated and analyzed during the current study are available from the corresponding author upon request. Data can also be found in the following link: https://drive.google.com/drive/folders/1Trb3-pbWI_ikq_XNO6taReaNk1C6dVoH?usp=sharing.

## Supporting Information Available

The effect of variable ML228 concentrations on collagen-I levels in HGF cells (Figure S1); the effect of variable ML228 concentrations on collagen-I levels in MG-63 osteoblast cells (Figure S2); and the evaluation of ML228 autofluorescence (Figure S3)(PDF)
