# Supplementary material for: Enhancement of Collagen-I Levels in Human Gingival Fibroblasts by Small Molecule Activation of HIF-1α
Source: J Agric Food Chem. 2023 May 3;71(20):7829–35. doi: 10.1021/acs.jafc.2c09059 (PMC10214441; doi:10.1021/acs.jafc.2c09059)
Supplement: Supplementary file 1 — jf2c09059_si_001.pdf [file jf2c09059_si_001.pdf]

Supplementary Information

Enhancement of collagen-I levels in human gingival fibroblasts by small molecule activation of HIF-1α

Lucia Adriana Lifshits[#], Miryam Rabin[#], Ran Tohar[#], Francesca Netti, Matan Gabay, Marina Sova, Daniel Z. Bar, Evgeny Weinberg, Lihi Adler-Abramovich, and Maayan Gal*

Department of Oral Biology, The Goldschleger School of Dental Medicine, Faculty of Medicine, Tel Aviv University, Tel Aviv 6997801, Israel

[#]Equally contributing first authors

***Corresponding author**

mayyanga@tauex.tau.ac.il; Tel.: +972-50-7987058

Figure S1-A-B shows confocal images taken 48 hours after treatment of HGF cells with variable concentrations of ML228 (representative concentrations are shown in Fig. 2). The maximal level of collagen is observed following treatment with 5 μM. Figure S1-C shows normalized collagen-I levels relative to non-treated cells.

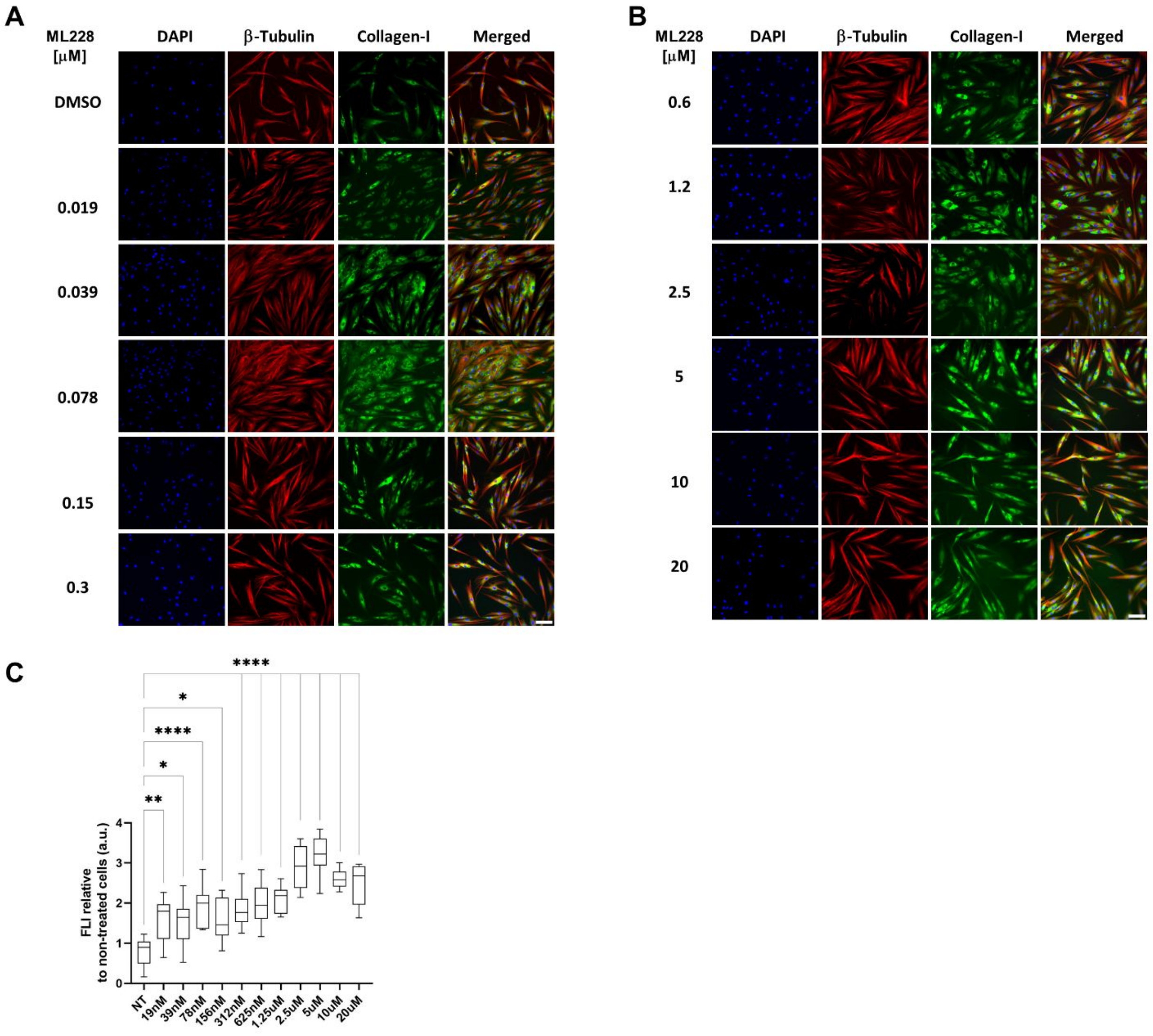

Figure S1. The effect of ML228 on collagen-I levels in HGF cells. Cells were cultured for 48 hours with variable concentrations of ML228 and stained against collagen-I, β-tubulin and DAPI (green,

red and blue, respectively). (A-B) Representative images of HGF cells treated with ML228. Scale bar: 100μm. (C) Mean normalized fluorescence intensity of collagen-I relative to non-treated cells. Images were taken by confocal microscopy and analyzed using Fiji 2 to evaluate fluorescence intensity. Values were measured using Color Threshold tab and normalized to the mean collagen intensity in the non-treated cells. Scale bar: 200μm

To explore the generality of collagen accumulation following HIF activation, we evaluated collagen levels in osteoblast MG-63 cells. Figure S2-A shows representative confocal images of collagen, β-tubulin and DAPI (green, red and blue, respectively) in the osteoblast cells. Figure S2-B shows a bar chart of the mean fluorescence intensity normalized to β-tubulin of MG-63 cells. As can be seen, the maximum enhancement of collagen-I at an ML228 concentration of 5 μM. To better understand the pattern of collagen accumulation following treatment with ML228, we further studied the effect of ML228 on cellular viability and proliferation. We applied the live/dead (green/red) viability assay as an orthogonal approach. Figure S2-C shows representative images of MG-63 cells treated with ML228 at 0, 0.62 and 5 μM. Images following treatment with 70% ethanol resembles cell death. The fluorescence images (merged in green and red) after incubation of the cells with ML228 for 48 hours show that ML228 negatively regulated cell growth without imparting cellular death. Figure S2-D shows the cellular viability (green) and cellular death (red) relative to non-treated cells.

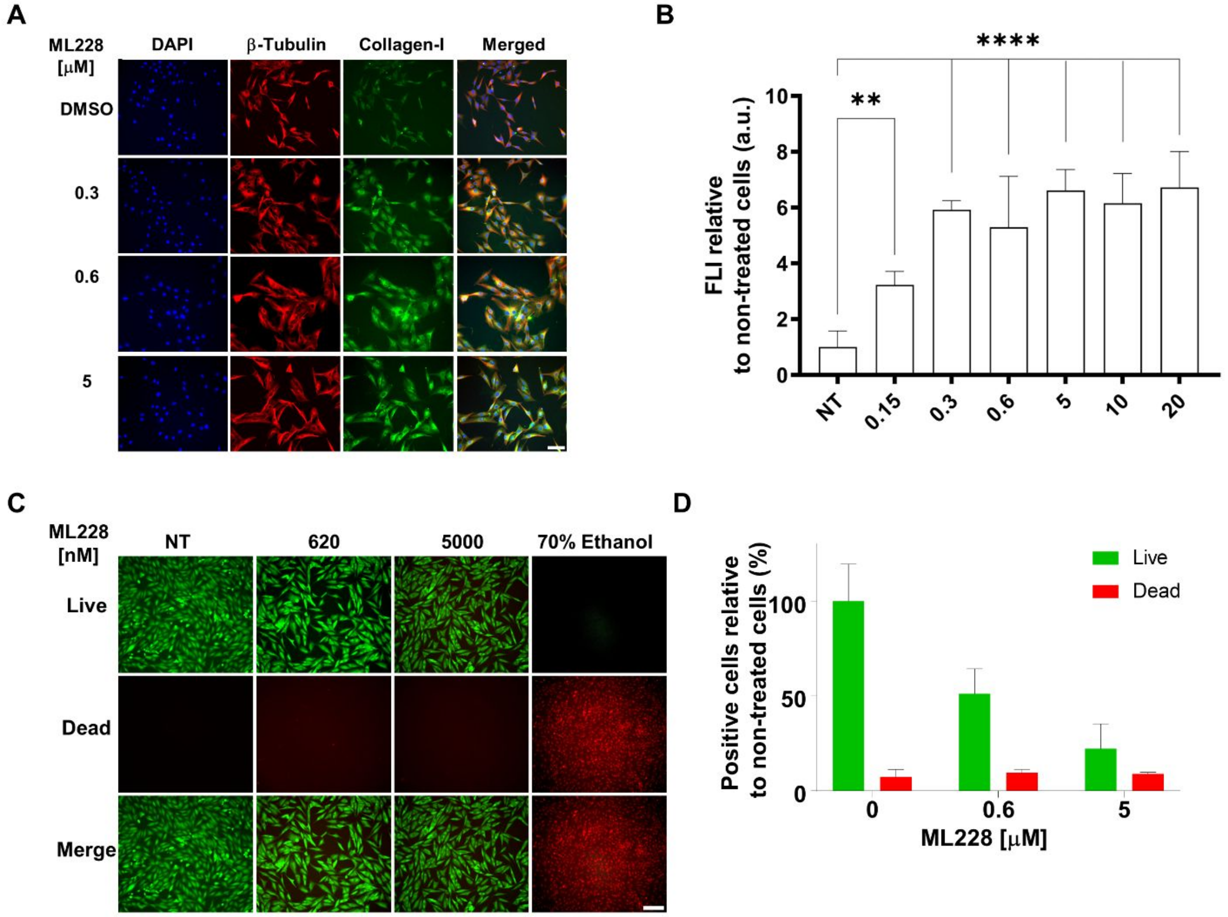

Figure S2. The effect of ML228 on collagen-I levels in MG-63 osteoblasts. Cells were cultured for 48 hours with variable concentrations of ML228, and stained with collagen-I antibody (green), β-tubulin (red) and DAPI (blue). (A) Representative confocal images of osteoblasts following treatment with ML228. Scale bar: 100μm. (B) Mean fluorescence intensity relative to non-treated cells and normalized relative to the number of cells, as a function of ML228 concentration. (C) Cellular viability assay of osteoblast cells treated with ML228. Cells were incubated with variable concentrations of ML228 for 48 hours, and cellular viability was evaluated via the live/dead assay. Representative confocal images (top) of live (green) and dead (red) cells treated with various concentrations of ML228, and with 70% ethanol as a reference for dead cells (right column). (D) Normalized fluorescence intensity of treated relative to non-treated cells.

Figure S3 shows blue, green and red images taken with the same parameters as described in Figure 1 but without fluorescence antibodies (primary and secondary as well). This concludes that ML228 does not impart any detectable autofluorescence.

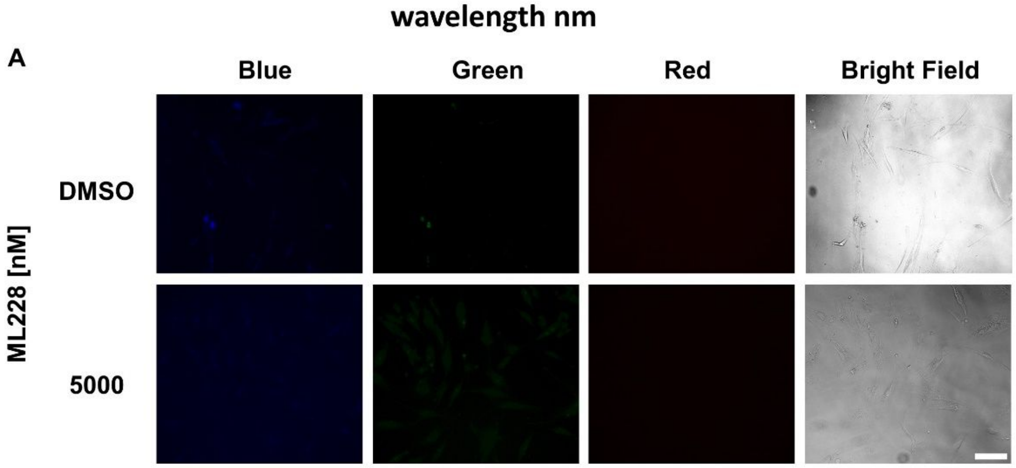

Figure S3. ML228 does not have autofluorescence. Immunofluorescence of non-treated (DMSO) and treated HGF cells with 5μM of ML228 without DAPI, β-tubulin and collagen-I antibodies. Scale bar: 100μm**.**

Methods

MG-63 human osteosarcoma cell lines were obtained from the American Type Culture Collection (ATCC, Manassas, VA, USA). Cells were cultured in α-MEM supplemented with 10% FCS, 100 U/ml penicillin and 100mg/ml streptomycin at 37°C at 5% CO_2. Before treatment with ML228, 5,000 and 50,000 cells/well were seeded in a 96 or 24 well-plate, respectively.
